# Lumbar and ventricular CSF concentrations of extracellular matrix proteins before and after shunt surgery in idiopathic normal pressure hydrocephalus

**DOI:** 10.1186/s12987-021-00256-1

**Published:** 2021-05-13

**Authors:** Karolina Minta, Anna Jeppsson, Gunnar Brinkmalm, Erik Portelius, Henrik Zetterberg, Kaj Blennow, Mats Tullberg, Ulf Andreasson

**Affiliations:** 1 Department of Psychiatry and Neurochemistry, Institute of Neuroscience and Physiology, the Sahlgrenska Academy At the University of Gothenburg, Sahlgrenska University Hospital/Mölndal, 431 80 Mölndal, Sweden; 2 Department of Clinical Neuroscience, Institute of Neuroscience and Physiology, the Sahlgrenska Academy At the University of Gothenburg, Mölndal, Sweden; 3 Clinical Neurochemistry Laboratory, Sahlgrenska University Hospital, Mölndal, Sweden; 4 Department of Neurodegenerative Disease, UCL Institute of Neurology, London, UK; 5 UK Dementia Research Institute At UCL, London, UK

**Keywords:** Brevican, Idiopathic normal pressure hydrocephalus, Immunoassay, Mass spectrometry, Matrix metalloproteinase, Neurocan, Shunt surgery, Tissue inhibitor of metalloproteinases

## Abstract

**Background:**

Idiopathic normal pressure hydrocephalus (iNPH) is a reversible CNS disease characterized by disturbed cerebrospinal fluid (CSF) dynamics. Changes in the extracellular matrix (ECM) composition might be involved in the pathophysiology of iNPH. The aim of this study was to explore possible differences between lumbar and ventricular CSF concentrations of the ECM markers brevican and neurocan, matrix metalloproteinases (MMPs) and tissue inhibitor of metalloproteinase-1 (TIMP-1) and their relation to clinical symptoms in iNPH patients before and after shunt surgery.

**Methods:**

Paired lumbar and ventricular CSF was collected from 31 iNPH patients, before and four months after shunt surgery. CSF was analysed for concentrations of tryptic peptides originating from brevican and neurocan using a mass spectrometry-based panel, and for MMP-1, -2, -9, -10 and TIMP-1 using fluorescent or electrochemiluminescent immunoassays.

**Results:**

Brevican and neurocan peptide levels were not influenced by CSF origin, but MMP-1, -2, -10 and TIMP-1 were increased (p ≤ 0.0005), and MMP-9 decreased (p ≤ 0.0003) in lumbar CSF compared with ventricular CSF. There was a general trend of ECM proteins to increase following shunt surgery. Ventricular TIMP-1 was inversely correlated with overall symptoms (rho = − 0.62, p < 0.0001). CSF concentrations of the majority of brevican and neurocan peptides were increased in iNPH patients with a history of cardiovascular disease (p ≤ 0.001, AUC = 0.84–0.94) compared with those without.

**Conclusion:**

Levels of the CNS-specific proteins brevican and neurocan did not differ between the lumbar and ventricular CSF, whereas the increase of several CNS-unspecific MMPs and TIMP-1 in lumbar CSF suggests contribution from peripheral tissues. The increase of ECM proteins in CSF following shunt surgery could indicate disturbed ECM dynamics in iNPH that are restored by restitution of CSF dynamics.

**Supplementary Information:**

The online version contains supplementary material available at 10.1186/s12987-021-00256-1.

## Background

Idiopathic normal pressure hydrocephalus (iNPH) is the dominating form of hydrocephalus in adults, characterized by disturbed cerebrospinal fluid (CSF) dynamics with enlargement of the entire ventricular system, but no macroscopic obstruction to CSF flow [1]. The clinical features of iNPH include gait abnormality, problems with balance, urinary urgency and incontinence, and cognitive impairment [1, 2].

Symptoms of iNPH can be reversed by diverting CSF, usually by a ventriculo-peritoneal or ventriculo-atrial shunt, where CSF is diverted from the cerebral ventricular system into the peritoneal cavity or the right atrium of the heart, respectively [2]. Shunt surgery in iNPH is considered to be a definite and cost-effective medical treatment [3] with significant improvement in up to 80% of the patients [4]. Nevertheless, many iNPH patients remain undiagnosed and undertreated [5, 6]. Although iNPH is characterized by changes in CSF dynamics, the underlying disease mechanisms remain largely unknown. CSF markers can serve as an ideal tool for diagnostic and predictive purposes in iNPH. Changes in concentrations of CSF biomarkers reflecting a variety of pathological changes in iNPH patients have been previously reported. These include elevated neurofilament light (NFL) levels indicating damage to large myelinated axons [7–9] and increased glial fibrillary acidic protein (GFAP) levels implying astrogliosis [8, 10], while decreased levels of products derived from amyloid precursor protein (APP) might be explained by the reduced brain metabolism in the periventricular zone [9, 11]. Also, a reduced flow in the interstitial fluid space and impaired glymphatic circulation have been suggested [12, 13]. In addition, the water content in the brain extracellular space is markedly increased in iNPH patients [14], possibly leading to reduced ECM flow. Shunt surgery has been shown to induce biochemical changes in CSF, such as elevation of APP-derived products (sAPPα, sAPPβ and amyloid-β peptides) [9], albumin, tau and some monoamine metabolites [15]. Several preoperatively investigated CSF markers did not show any differences between patients that improved or did not improve after surgery, although high preoperative CSF NFL levels correlated with more favourable outcome after surgery [7, 8, 11]. There is still a great need for reliable markers for iNPH outcome prediction following shunt surgery. Identifying CSF-based markers with such an ability would aid in the selection of patients who could benefit from this procedure.

The differences in biochemical composition and dynamics between the two origins of CSF (lumbar and ventricular) are still largely unexplored, mainly because of ethical considerations regarding collection of ventricular CSF. As CSF travels from the ventricles to the spinal channel, there is a general progressive increase in cell count and total protein concentration in lumbar CSF with increasing distance from the brain [16, 17]. In addition, CSF absorption occurs mostly along cerebral convexities, either from the subarachnoid space to the venous system (across the arachnoid villi) or from the cerebral ventricular walls into the brain parenchyma (at the site of periventricular white matter) [18]. However, considerable portions of CSF may be absorbed into the cervical lymph nodes, nasal lymphatics or parenchymal capillaries of spinal cord [18–20]. It is generally accepted that the total protein content of lumbar CSF is 60% higher than that of ventricular CSF [16]. However, some proteins, *e.g.*, tau, were reported to be higher in ventricular CSF than in lumbar CSF [15].

The appropriate assembly of brain extracellular matrix (ECM) is essential for maintaining a suitable environment for cellular functions. The two CNS-specific ECM proteoglycans, brevican and neurocan, are uniquely expressed in brain and spinal cord, and are, together with tenascins and hyaluronic acid the main modulators of brain plasticity by regulating cell migration and axonal growth [21]. Proteoglycans are the major substrates for several matrix metalloproteinases (MMPs), belonging to a 23-member family of zinc-dependent endopeptidases that are responsible for both physiological and pathological tissue remodelling [22]. The MMPs are divided into several subgroups based on substrate specificity and structural similarities, including collagenases (MMP-1), gelatinases (MMP-2 and -9) or stromelysins (MMP-10). Tissue inhibitor of metalloproteinases-1 (TIMP-1) is capable to inhibit the activity of most of the MMPs, although the efficacy of this inhibition varies for each MMP [23]. MMPs and TIMP-1 are non-CNS specific and play crucial roles in immune responses and their dysregulation occurs in many inflammatory and autoimmune diseases [22, 24].

The ECM composition is tightly regulated under physiological conditions. Disruption of the balance between the production of ECM proteins as well as their proteolytic processing (by MMPs) and processing inhibition (by TIMPs) may result in pathological conditions associated with uncontrolled ECM turnover [25]. The iNPH pathophysiology seems to affect the ECM composition in CSF [26, 27], possibly reflecting astroglial activation in the iNPH patients [8, 10], although additional research is highly warranted. In addition, increased water content in the brain extracellular space might be a main cause of reduced ECM flow in iNPH patients [14]. Thus, in iNPH, ECM metabolism is likely to be modified and its dynamics, including the ECM flow, might be improved after shunt surgery.

The primary aim of this study was to explore the biochemical ECM changes in iNPH at baseline and after shunt surgery in both lumbar and ventricular CSF, and in relation to clinical symptoms. In addition, lumbar and ventricular CSF were compared in the context of ECM protein concentrations. To our knowledge, this is the first study on iNPH patients evaluating ECM components in both lumbar and ventricular CSF, in relation to shunt surgery.

## Methods

### Patient characteristics

The study cohort consisted of 31 patients [22 male and 9 female, 74 ± 7 years (mean ± SD)] diagnosed with iNPH according to international criteria [1] at the Hydrocephalus Research Unit, Sahlgrenska University Hospital, Gothenburg, Sweden (see Table 1 for the patients’ clinical characteristics). A pathologic ventricular enlargement was estimated using Evans’ index value of > 0.3, defined as the ratio between the maximal width of the frontal horns and the maximal internal diameter of the skull [28]. Patients were examined clinically pre- and four months postoperatively by a neurologist, a physiotherapist and a neuropsychologist. Severity of symptoms was scored on the iNPH scale introduced by Hellström et al*.* [29], composed of items assessing four domains—gait, balance, cognition and continence—yielding a score of 0–100 in each domain, 0 referring to maximal symptoms and 100 to no symptoms, and a total score of 0–100 of the combined domain scores. Outcome was defined as the difference between the post- and the preoperative iNPH scale score, *i.e.*, a positive value indicating improvement, the higher the positive value, the better the improvement. Patients were classified as either improved if the postoperative change in iNPH scale was ≥ 5 points, or unimproved (change of < 5 points) [29].

**Table 1:** Clinical characteristics of 31 iNPH patients at baseline, and pre- and postoperative iNPH scale scores

Clinical characteristics	Value
Age (mean ± SD)	74 ± 7
Gender (male/female)	22/9
Symptom duration in months (mean ± SD)	44 ± 40
Diabetes (%)	42
Hypertension (%)	61
Cardiovascular disease (%)	29
MMSE (median, IQ interval)	27 (24–28)
Daily need of sleep in hours (mean ± SD)	9 ± 3
iNPH scale score	
Baseline (median, IQ interval)	56 (50–74)
Postoperative (median, IQ interval)	70 (53–81)

*MMSE* mini mental state examination

Cardiovascular disease: atrial fibrillation, ischemic heart disease, congestive heart disease

All patients received a ventriculo-peritoneal shunt with a programmable valve (PS Medical Strata, Medtronic Inc., Goleta, CA, USA) with an anti-siphon device and a Rickham reservoir. Three patients were excluded from the postoperative clinical outcome assessment due to development of symptoms caused by another progressive disorder (multiple sclerosis, amyotrophic lateral sclerosis or disseminated malignant disease). All patients had working shunts and none had complications at the time for follow-up evaluation.

Preoperative lumbar CSF (12 mL) was sampled in the morning at the routine preoperative examination in the L3/L4 or L4/L5 interspace with the patient in a recumbent position. Preoperative ventricular CSF was sampled through the catheter introduced in the right lateral ventricle at the time for shunt surgery: the first 2 mL of CSF were discarded, and the next 8 mL were collected. Postoperative ventricular CSF (8 mL) was sampled at the four months’ postoperative re-examination via a puncture of the Rickham reservoir followed by sampling of postoperative lumbar CSF as described above. No infection or other complication occurred due to the post-operative Rickham reservoir puncture. All CSF samples were centrifuged, aliquoted and kept at -80 °C until analysed.

Longitudinal samples from the same patient were placed next to each other on the same plates to reduce the effect of possible inter-plate variation. Study samples were run in singlicate, but analytical variation was monitored using CSF pool quality samples.

### Brevican/neurocan analysis

The brevican/neurocan panel has previously been described in detail [30]. Briefly, in total 20 isotope-labelled tryptic peptides (nine for brevican and eleven for neurocan), labelled with ^13^C and ^15^ N at the C-terminal arginine or lysine, were used as internal standard (IS) peptides (JPT Peptide Technologies, Berlin, Germany) (Additional file 1: Table S1). Twenty-five μL of the IS mixture was spiked into 100 μL CSF, followed by reduction, alkylation, trypsin digestion and subsequent sample clean-up.

Next, the dried samples were reconstituted in 100 µL 50 mM ammonium bicarbonate. Samples were then analysed by high-performance liquid chromatography (HPLC)—mass spectrometry (MS)-based method with a parallel reaction monitoring (PRM) approach, using a Vanquish UHPLC coupled to a high resolution Q Exactive hybrid quadrupole-orbitrap mass spectrometer, with electrospray ionization (Thermo Fisher Scientific), operated as previously described [30, 31]. Each sample (50 µL) was loaded onto a Hypersil Gold reversed phase HPLC C18 column (Thermo Fisher Scientific) operated at a flow rate of 300 µL/min. Mobile phases were A: 0.1% formic acid in deionized water (v/v) and B: 0.1% formic acid/84% acetonitrile in deionized water (v/v/v). The applied gradient was 0 to 40% B over 20 min.

### TIMP-1 analysis

TIMP-1 concentrations in CSF were measured using a singleplex electrochemiluminescent immunoassay (K151JFC, MSD Multi-Array, Meso Scale Discovery, Rockville, MD, USA), following the manufacturer’s instructions.

### MMP panel analysis

MMPs (MMP-1, -2, -9 and -10) concentrations in CSF were measured using a Milliplex MAP Human MMP magnetic bead panel (HMMP2MAG-55 K, EMD Millipore Corp., Billerica, MA, USA), following the manufacturer’s instructions.

### Statistical analysis

Apart from MMP-2, none of the analytes were normally distributed based on the Kolmogorov–Smirnov test. Changes between the pre- and postoperative measurements in CSF of two different origins (lumbar and ventricular) were analysed using Friedman test with Dunn’s multiple comparisons. Mann–Whitney test or Kruskal–Wallis test with Dunn’s multiple comparisons were applied to investigate the differences between two or more independent groups of various clinical characteristics (clinical outcome after shunt surgery, cardiovascular disease, diabetes and/or hypertension), respectively. To evaluate if the patients were age- and gender-matched, Mann–Whitney test or Chi-square test were applied, respectively. Area under the curve (AUC) from receiver operating characteristic analysis was used as a measure of the effect size. Spearman correlation coefficients were calculated in correlation analyses. A p-value of less or equal to 0.002, after adjustment using Bonferroni correction for multiple testing (n = 23), was considered significant. Statistical analyses were performed using GraphPad Prism, version 7.03 (GraphPad Software, Inc., San Diego, CA, USA) and SPSS software, version 26 (IBM Corp., Armonk, NY, USA).

### Validation

For the brevican/neurocan panel, four replicates each of two different CSF pool quality samples were evenly spread out throughout the 96-well plate. For the MMP panel and TIMP-1 assay, the two different CSF pool quality samples were placed at the beginning and end of the 96-well plate. The intra- and inter-assay variabilities were determined by calculating the coefficient of variation (CV) for these replicates. A more extensive validation of the in-house brevican/neurocan panel was performed previously [30]. Briefly, the majority of brevican and neurocan peptides in CSF showed analytical stability for up to five freeze–thaw cycles and storage stability under different conditions: −80 °C for one month, − 20 °C for one month, 5–8 °C for 24 h, 5–8 °C for 7 days, and room temperature for 24 h. In addition, the relative error of the back-calculated concentrations were below 20% for the majority of calibrators, except for N1195.

### Data availability

The data supporting the findings in this study are available from the corresponding author, upon reasonable request.

## Results

### Lumbar vs. ventricular CSF

**Fig. 1:**
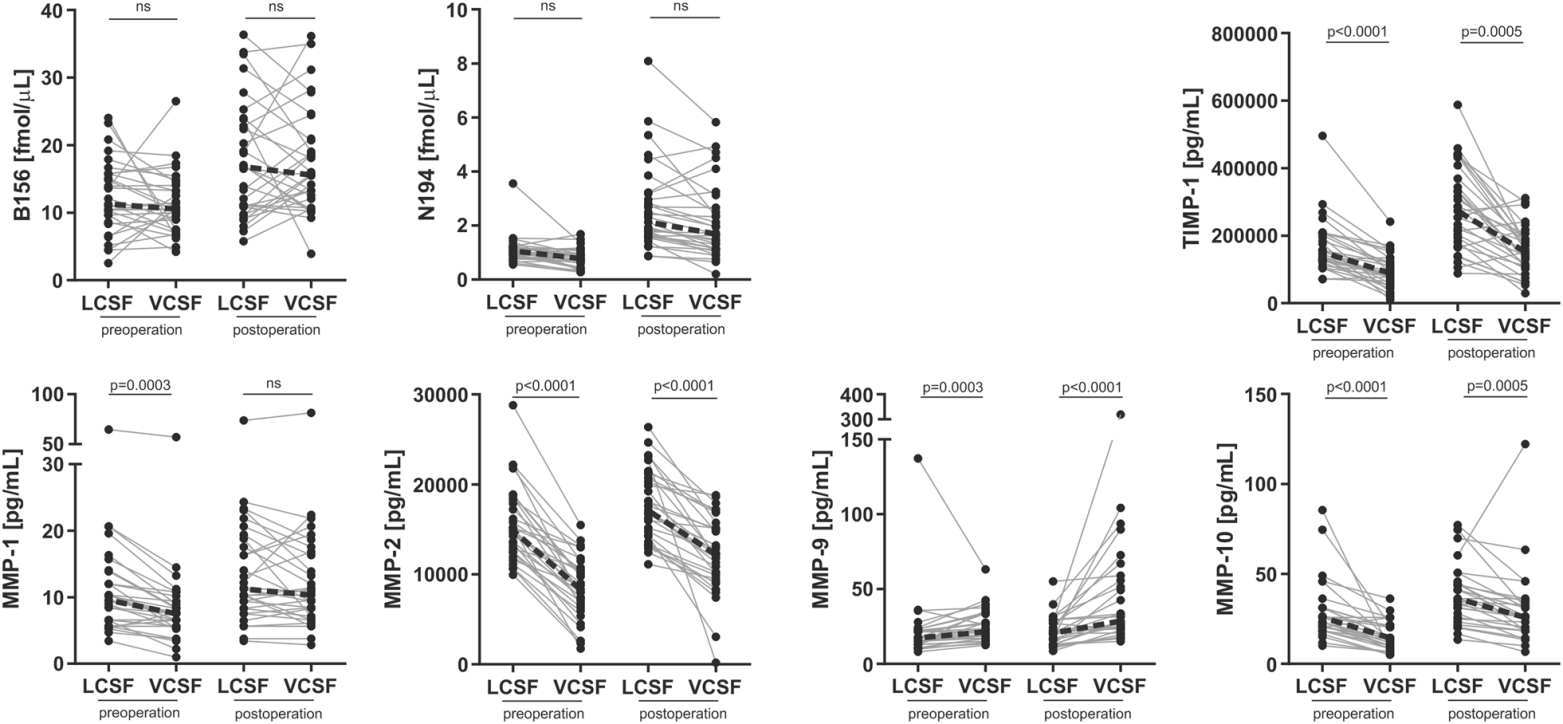
CSF concentrations of ECM proteins between lumbar and ventricular CSF before and after shunt surgery. The CNS-specific brevican and neurocan peptide concentrations did not differ between the lumbar and ventricular CSF (both pre- and post-operation), while the majority of MMPs and TIMP-1 concentrations were increased in lumbar CSF compared with ventricular CSF. MMP-9 showed opposite dynamics—its concentrations were higher in ventricular CSF compared with lumbar CSF. Friedman test with Dunn’s multiple comparisons and Bonferroni correction for multiple testing were applied to investigate the differences between the lumbar and ventricular CSF. A representative peptide of brevican (B156) and of neurocan (N194) has been displayed (see Additional file 1: Fig. S1 for all peptides). Dotted line represents change in median. *LCSF* lumbar CSF, *VCSF* ventricular CSF

The brevican and neurocan peptide concentrations did not differ between the lumbar and ventricular CSF, neither pre- nor post- operatively (p = 0.003–0.99) (Fig. 1 and Additional file 1: Fig. S1). The concentrations of MMP-1, -2 and -10 were higher in lumbar CSF compared with ventricular CSF before shunt surgery (all three MMPs) and after (MMP-2 and -3) (p ≤ 0.0005), whereas MMP-9 showed the opposite pattern—its levels were elevated in ventricular CSF both pre- and postoperatively (p ≤ 0.0003) (Fig. 1). The lumbar CSF concentrations of TIMP-1 were higher compared with ventricular CSF in iNPH patients before and after shunt surgery (p ≤ 0.0005) (Fig. 1). The majority of CSF brevican and neurocan peptides levels correlated with each other, although this correlation was much stronger in ventricular CSF compared with lumbar CSF (Additional file 1: Fig. S2).

### Pre- vs. postoperative differences

**Fig. 2:**
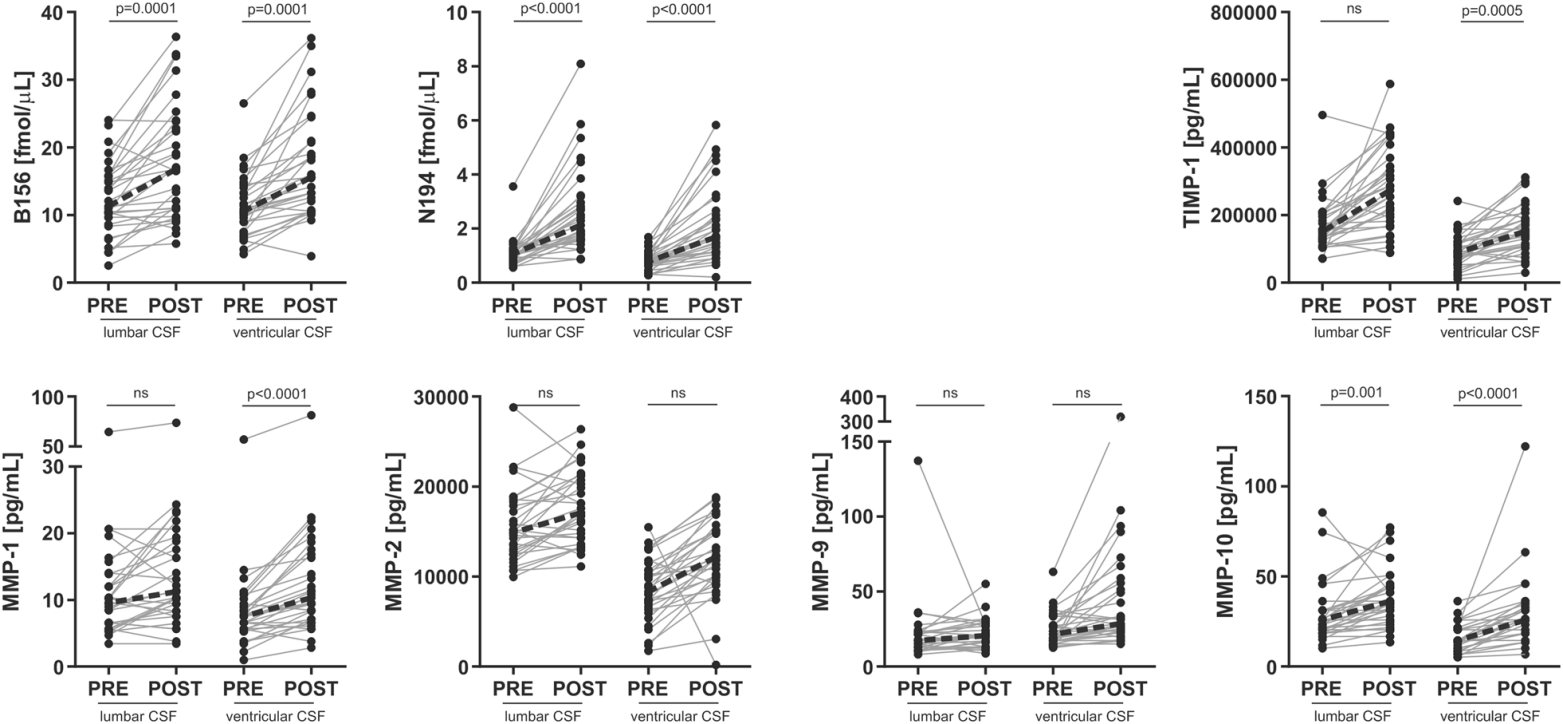
Lumbar and ventricular CSF concentrations of ECM proteins pre- and postoperatively in 31 iNPH patients. There was a general trend of ECM proteins to increase following shunt surgery, in both lumbar and ventricular CSF. Friedman test with Dunn’s multiple comparisons and Bonferroni correction for multiple testing were applied to investigate the differences before (``PRE``) and after (``POST``) shunt surgery. Dotted line represents change in median. A representative peptide of brevican (B156) and of neurocan (N194) has been displayed (see Additional file 1: Fig. S3 for all peptides)

The tryptic peptide levels of brevican and neurocan increased in both lumbar and ventricular CSF following shunt surgery compared with preoperative levels (p ≤ 0.002) (Fig. 2 and Additional file 1: Fig. S3). However, based on the correlation matrices, there was no clear change in brevican/neurocan fragment pattern after shunt surgery (Additional file 1: Fig. S2). Ventricular CSF MMPs and TIMP-1 showed a postoperative increase, either significant or at trend level (p < 0.0001–0.04), whereas the postoperative changes in lumbar CSF were less clear (p = 0.001–0.57) (Fig. 2).

### Associations between ECM protein concentrations and clinical symptoms and postoperative outcome

**Fig. 3:**
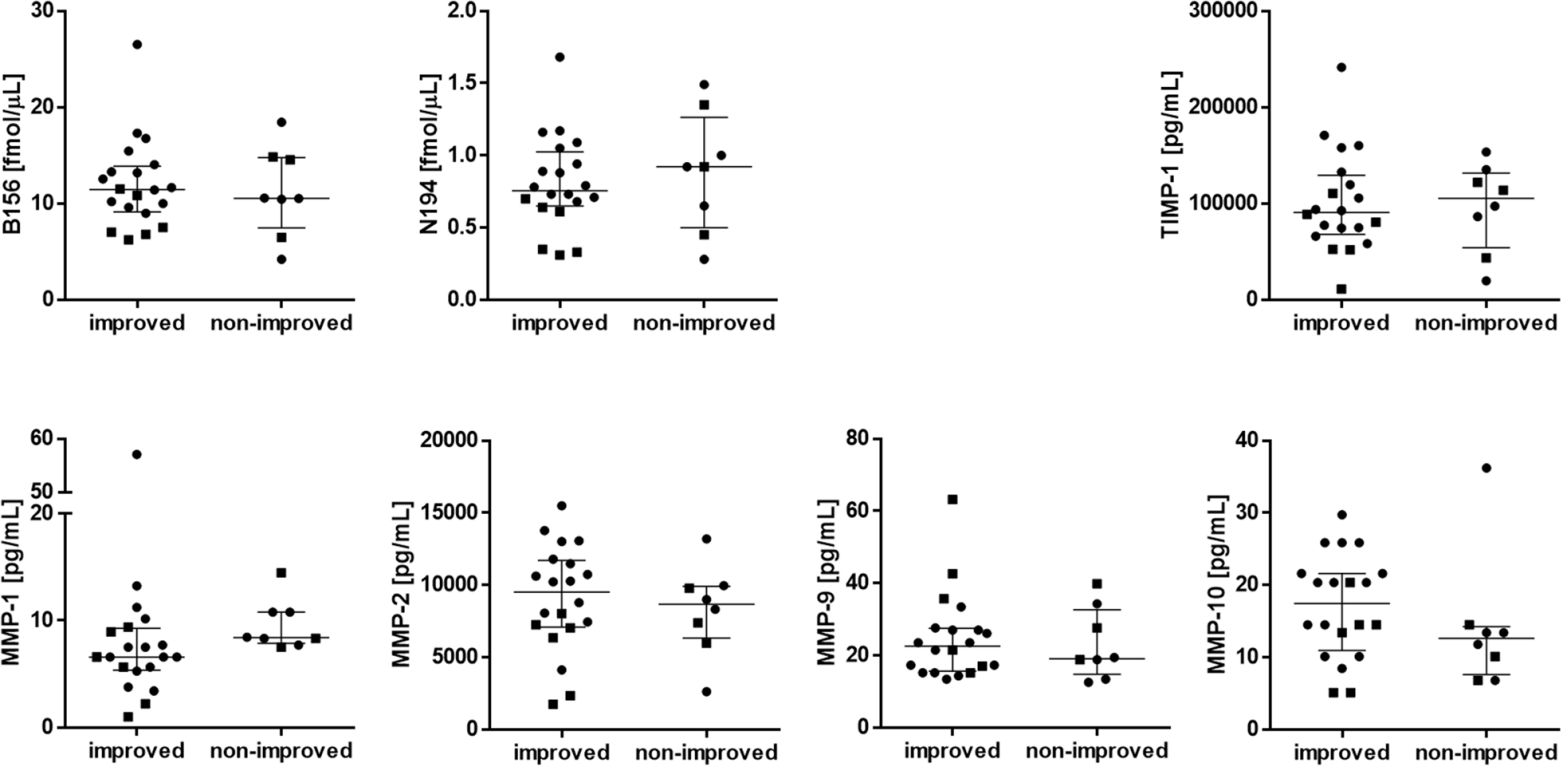
Preoperative ventricular CSF concentrations of ECM proteins in relation to clinical outcome after shunt surgery. None of the ECM proteins showed any differences between patients that improved after surgery and those who remained unchanged. Mann–Whitney test was applied to investigate the differences between the patients who did (improved; n = 20) or did not (non-improved; n = 8) improve following shunt surgery. INPH patients without any co-morbidities are displayed in the graphs as squares. The two groups were age- and gender-matched. A representative peptide of brevican (B156) and of neurocan (N194) has been displayed

**Fig. 4:**
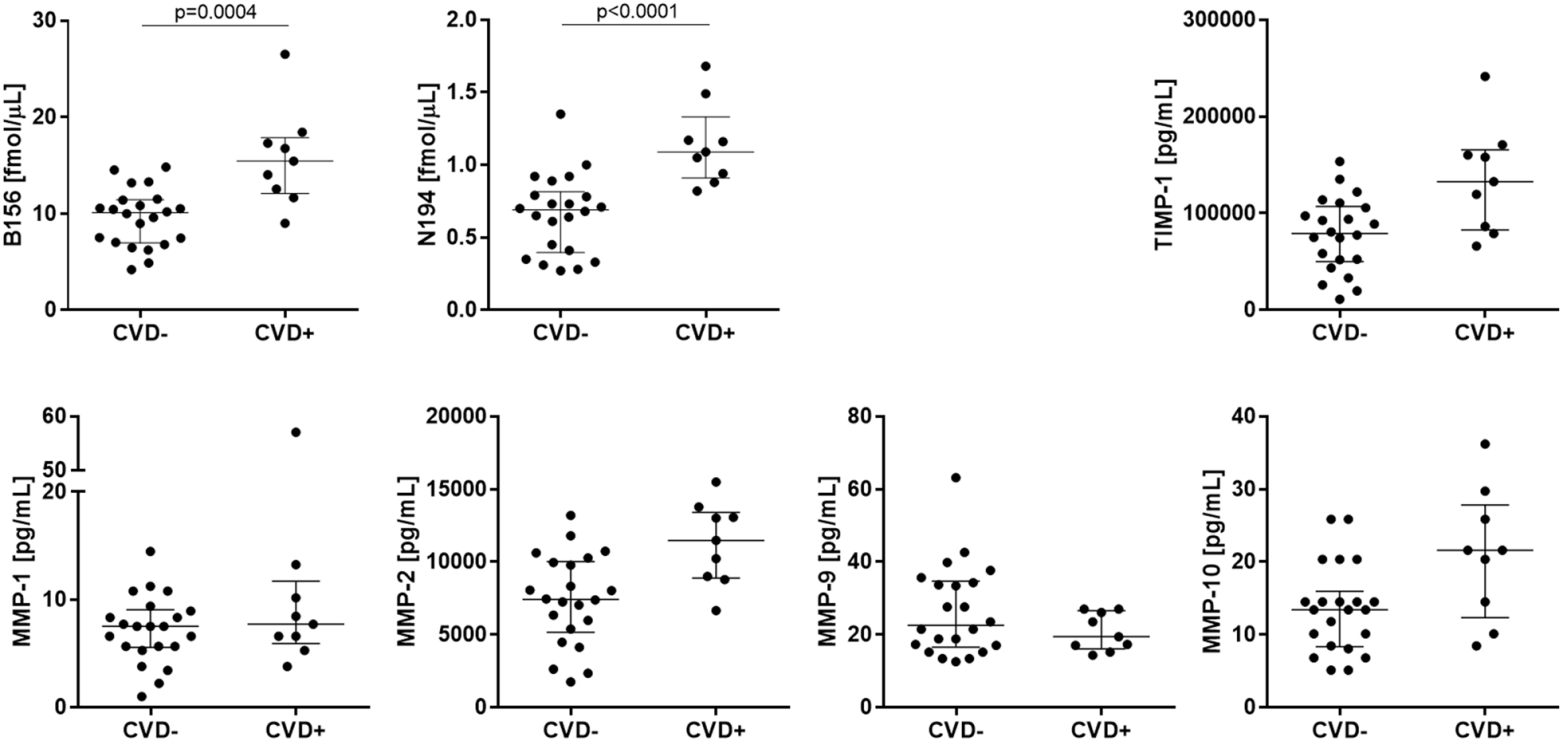
Ventricular, preoperative CSF concentrations of ECM proteins in relation to cardiovascular disease comorbidity as a single risk factor. Brevican and neurocan peptide levels were increased in patients who suffered from CVD comorbidity. Mann–Whitney test was applied to investigate the differences between the patients who did (CVD + ; n = 9) and did not (CVD-; n = 22) suffer from cardiovascular disease (CVD). The two groups were age- and gender-matched. A representative peptide of brevican (B156) and of neurocan (N194) has been displayed (see Additional file 1: Fig. S3 for all peptides)

At baseline, TIMP-1 was negatively correlated to total iNPH scale score (rho = − 0.62, p < 0.0001), otherwise there were no or weak correlations between the CSF ECM protein concentrations and the total iNPH scale score or the gait, balance, cognition or continence domain scores (Table 2). In addition, there was no association between the CSF ECM protein concentrations and a measure of ventriculomegaly (Evans’ index) (Table 2). None of the ECM proteins in preoperative lumbar or ventricular CSF showed any differences between patients that improved (n = 20) after surgery and those who remained unchanged (n = 8) (Fig. 3). Patients who suffered from cardiovascular comorbidity (atrial fibrillation, ischemic heart disease, congestive heart disease) had increased ventricular, but not lumbar, preoperative CSF concentrations of the majority of brevican and neurocan peptides compared with patients without (p ≤ 0.001, AUC = 0.84–0.94) (Fig. 4 and Additional file 1: Fig. S4).

**Table 2:** Correlations of ECM protein levels with various clinical variables in preoperative lumbar and ventricular CSF

	B156	N194	MMP-1	MMP-2	MMP-9	MMP-10	TIMP-1
Lumbar							
Age	− 0.15	0.37	0.41	0.48	0.15	-0.27	0.45
Symptom duration	− 0.09	0.07	− 0.04	0.25	− 0.22	0.27	0.31
Gait domain	0.26	0.09	0.01	− 0.38	0.01	− 0.25	− 0.37
Balance domain	0.18	0.04	0.21	− 0.25	− 0.15	− 0.08	− 0.41
Cognition domain	0.62*	0.41	− 0.18	− 0.41	0.15	− 0.28	− 0.39
Continence domain	− 0.06	0.13	0.42	0.45	0.28	− 0.16	0.31
Total inph scale	0.31	0.12	0.03	− 0.39	0.22	− 0.26	− 0.43
Daily need of sleep in hours	0.08	0.07	− 0.23	− 0.16	− 0.14	− 0.29	0.06
ΔiNPHscale score	− 0.05	− 0.25	− 0.32	− 0.09	0.00	0.11	0.02
Evans’ index	0.03	0.08	− 0.06	0.18	0.05	− 0.12	0.29
Ventricular							
Age	− 0.05	0.15	0.31	0.11	0.00	− 0.30	0.57*
Symptom duration	0.37	0.52	0.02	0.45	− 0.21	0.46	0.44
Gait domain	0.26	0.19	0.06	− 0.16	0.14	0.06	− 0.27
Balance domain	0.20	0.00	0.22	− 0.11	− 0.04	0.05	− 0.15
Cognition domain	0.12	0.02	− 0.36	− 0.33	0.18	0.01	− 0.54*
Continence domain	0.13	0.12	0.40	0.14	0.18	− 0.18	0.26
Total inph scale	0.06	− 0.15	− 0.32	− 0.44	0.37	− 0.11	− 0.62*
Daily need of sleep in hours	0.18	0.16	− 0.09	0.16	− 0.30	0.00	0.17
ΔiNPHscale score	− 0.17	− 0.29	− 0.46	− 0.07	0.05	0.08	− 0.11
Evans’ index	0.37	0.50	0.03	0.39	− 0.11	0.14	0.21

Spearman correlation coefficients were calculated in correlation analyses and rho values are displayed. Results with p < 0.002 are marked with an asterisk (*). A representative peptide of brevican (B156) and of neurocan (N194) has been included. The other brevican/neurocan peptides showed correlations very similar to the representative peptides. Gait, balance, cognition and continence domain refers to iNPH scale domains

ΔiNPH scale score refers to the postoperative change in total iNPH scale score

A history of diabetes or hypertension did not have any significant effect on the ECM protein concentrations in CSF (Supplementary figs. 5 and 6), but there was a gradual trend of brevican/neurocan levels to increase with the number of vascular risk factors present, although the change did not reach significant level (p = 0.06–0.25) (Additional file 1: Fig. S7). The two independent patients groups, *e.g.*, improved vs unimproved patients, did not differ in age or gender.

### Precision

The intra- and inter- assay variabilities of brevican and neurocan peptides, MMP-1, -2, -9 and TIMP-1 measurements in two different CSF pool quality samples were below or equal to 20%. Although the intra-assay variability of MMP-10 measurements were 3–4% in both CSF pool quality samples, the inter-assay variation was higher (27–28%). As the paired samples from the same individual were placed in the same plate, the high inter-assay variability does not affect within individual comparisons. The B834 and N1242 peptides were excluded from the analysis due to a large degree of both intra- and inter-assay variability (CV > 40%).

## Discussion

To our knowledge, this is the first study on iNPH patients evaluating ECM components in both lumbar and ventricular CSF, in relation to shunt surgery. The sparse associations between CSF ECM protein levels and measures of the patients’ clinical symptomathology and the lack of associations with outcome following shunt surgery suggest that the concentration patterns of these ECM proteins are not primarily related to core pathophysiological mechanisms or reversibility in iNPH, and therefore not reliable markers for outcome prediction, but probably rather associated with more general or secondary processes. The negative correlations found between TIMP-1 and measures of cognitive and overall performance are interesting but require further exploration in larger sample sets or need to be repeated. The lack of associations between the CSF ECM proteins and degree of ventriculomegaly is consistent with the result from a previous study [32] where several other CSF biomarkers also did not correlate with the ventricular CSF volume.

This paper presents an interesting discrepancy between the concentrations of CNS-specific and -unspecific components of ECM in two different sources of CSF: lumbar and ventricular. The CNS-specific brevican and neurocan were not influenced by CSF origin, which indicates that their levels can be evaluated using the less invasive lumbar sampling for future research. However, the majority of MMPs as well as TIMP-1, that are widely expressed in a multiple range of tissues, were higher in lumbar CSF compared with ventricular CSF. The higher protein levels in lumbar CSF vs. ventricular CSF was previously observed for two other blood-derived proteins, IgG and albumin, and was explained either by their local synthesis in the subarachnoid space or increased endothelial cell permeability [16, 33, 34]. The elevated lumbar CSF levels of MMP-1, -2, -10 and TIMP-1 might also be caused by their potential release to the subarachnoid space from periphery along the spine. Interestingly, MMP-9 showed different, and difficult to explain, dynamics compared with the other MMPs. Its CSF levels were decreased in lumbar CSF compared with ventricular CSF. The CSF MMP-9 levels did not correlate with any of the other studied ECM proteins indicating that MMP-9 might measure different pathological changes in iNPH. In a previous study [27] we reported that MMP-9 did not strongly correlate neither to other MMPs nor to brain injury markers. In addition, MMP-9 seems to have different substrate preferences compared with other MMPs. It did not show any potential to degrade brevican, while MMP-1, -2 and -10 did [35, 36]. Neurocan also cannot be degraded by MMP-9, while MMP-2 showed this potential [35].

There is a general trend of ECM proteins to be elevated in CSF following shunt surgery. The comparatively high preoperative levels of brevican and neurocan in CSF was previously explained to possibly reflect chronic stages of iNPH, *e.g.*, astroglial activation [26]. Interestingly, shunt surgery has also been shown to lead to an increase in the CSF levels of markers for other pathologies such as NFL, APP-derived products, p-tau and albumin [9]. Shunt surgery improves the clinical symptoms in the vast majority of patients with iNPH [4] and thus, the observed overall postoperative increase of ECM proteins in CSF reported here might be a result of improved or normalized CSF flow and reversed iNPH pathophysiology. Speculatively, ECM proteins might be more easily released from the brain tissue to the ventricles as a result of improved CSF dynamics with reduction of ICP pulsations [37], decompression of the brain, improved periventricular white matter extracellular flow and better clearance of protein fragments into the CSF [38–41]. Interestingly, the non-CNS-specific part of ECM (MMPs and TIMP-1) showed an increase following operation mostly in ventricular CSF, while these changes were only partially reflected in lumbar CSF. The peripheral origin of MMPs and TIMP-1 might contribute to the lack of a clear increase in lumbar CSF following the shunt surgery. However, their increased levels in ventricular CSF following shunt surgery might be a result of improved CSF dynamics, as these ECM proteins are also expressed in the brain, in addition to other tissues.

The selection of ECM proteins was based on previous results, where CSF brevican and neurocan concentrations were indicated to be involved in the pathological processes of iNPH [26]. Moreover, CSF MMP-1, -2, -9 and -10 exhibited different dynamics in iNPH [27] compared with patients following traumatic brain injury. To our knowledge, CSF TIMP-1 levels have not been reported before in regard to iNPH. The reason for analysis of various brevican and neurocan fragments, not total proteins, was to evaluate if their fragmentation patterns and consequently the concentration of proteolytic break-down products are affected by shunt surgery. The previous study [42] showed that various brevican fragments might reflect different pathological changes in the brain, *e.g.*, the two peptides, B741 and B834, but not others, showed different dynamics and were significantly correlated to S100B. Thus, they might serve as potential indicators for astroglial pathophysiology. Although all the brevican and neurocan peptides correlated with each other in the iNPH group in the previous study [42], it would still be interesting to evaluate if the dynamics of individual peptides differ in longitudinal data of pre/post surgery. However, no discrepancy between the peptides was observed in the current study. Correlations between the brevican and neurocan peptides indicate that there is no evident dysregulated proteolytic processing of these proteins along their protein core in iNPH, and it is not affected by shunt surgery. Stronger correlations in ventricular CSF than in lumbar CSF might be explained by the CNS-specific origin of both proteoglycans and that ventricular CSF reflects more pure CNS pathophysiology. MMPs and TIMP-1 are abundantly present in various non-CNS tissues, which can explain that there is no clear difference in their correlations with each other between the two CSF origins.

We recently reported that CSF brevican and neurocan peptide concentrations were decreased in vascular dementia compared with healthy controls [30], and thus it was of interest to explore their levels in iNPH patients who showed vascular-based pathology. This study shows that ventricular CSF concentrations of brevican and neurocan peptides show a potential to distinguish iNPH patients who suffer from cardiovascular disease, and thus, might hypothetically serve as novel biomarker candidates to reflect cardiovascular disease related brain abnormalities in iNPH. We suggest that future studies explore this notion further and include associations between CSF brevican and neurocan peptides and measures of white matter hyperintensities in patients with iNPH. Moreover, since CSF brevican and neurocan peptide levels were decreased in vascular dementia compared with healthy controls [30], and thus might serve as potential diagnostic biomarkers, the even lower levels of these peptides in iNPH without cardiovascular comorbidities, might reflect further, different pathological processes specific to iNPH. However, as the two ECM proteins in iNPH have not been studied in comparison with healthy controls, it is difficult to evaluate the specificity of brevican/neurocan to diagnose iNPH when compared with its diagnostic ability for vascular dementia. Further studies should explore the association of the markers to radiological biomarkers of subcortical damage and/or white matter damage.

The strengths of this study include a well characterized patient group with detailed clinical assessment of symptoms and the collection of paired lumbar and ventricular CSF before and after shunt surgery for each patient.

However, there are some limitations to the study that should be acknowledged, including lack of healthy individuals in the cohort. Without a control group, it is not possible to fully evaluate the ECM changes involved in pathological processes specific for iNPH. In addition, the samples were not normalized to the total protein content. However, such normalization is not common practice for most immunoassays and not for targeted MS assays with internal standards. Another limitation is the low number of cases in the separate clinical groups as well as in the combined groups, which introduces a risk that associations or differences between groups remain undetected.

## Conclusions

In conclusion, CSF ECM protein levels are not reliable markers for outcome prediction after shunt surgery in iNPH. However, this study demonstrated prominent biochemical changes in ECM between lumbar and ventricular CSF of iNPH patients induced by shunt surgery. While brevican and neurocan concentrations are not affected by CSF origin, the majority of MMPs and TIMP-1 are elevated in lumbar CSF compared with ventricular CSF, suggesting contribution from peripheral tissues to the lumbar CSF levels of these proteins.

## Supplementary Information

**Additional file 1.** Additional figures and table.

## Data Availability

The datasets used and analysed during the current study are available from the corresponding author on reasonable request.

## Abbreviations

APP: Amyloid precursor protein
AUC: Area under the curve
CVD: Cardiovascular disease
CSF: Cerebrospinal fluid
CV: Coefficient of variation
EVD: External ventricular drain
ECM: Extracellular matrix
iNPH: Idiopathic normal pressure hydrocephalus
IS: Internal standard
MMP: Matrix metalloproteinase
MS: Mass spectrometry
MMSE: Mini-Mental State Examination
NFL: Neurofilament light
PRM: Parallel reaction monitoring
TIMP: Tissue inhibitor of metalloproteinases
