# Supplementary material for: Lumbar and ventricular CSF concentrations of extracellular matrix proteins before and after shunt surgery in idiopathic normal pressure hydrocephalus
Source: Fluids Barriers CNS. 2021 May 13;18:23. doi: 10.1186/s12987-021-00256-1 (PMC8120927; doi:10.1186/s12987-021-00256-1)
Supplement: Supplementary file 1 — Additional file 1. Additional figures and table. [file 12987_2021_256_MOESM1_ESM.docx]

**Supplementary table 1.** The peptides used in the PRM-MS assay with acquisition characteristics.

| **Name** | **Position** | **Sequence** | ***m/z***^a^ | **Charge, z** | **NCE^b^** |
| --- | --- | --- | --- | --- | --- |
| B87 | 87-94 | EAEVLVAR | 443.75 | 2 | 15 |
| B156 | 156-162 | GVVFLYR | 427.25 | 2 | 15 |
| B268 | 268-274 | LTLEEAR | 416.23 | 2 | 15 |
| B313 | 313-321 | YPIVTPSQR | 530.79 | 2 | 22 |
| B322 | 322-330 | CGGGLPGVK | 422.72 | 2 | 22 |
| B718 | 718-738 | MYGAHLASISTPEEQDFINNR | 798.38 | 2 | 22 |
| B741 | 741-750 | EYQWIGLNDR | 647.32 | 2 | 15 |
| B834 | 834-841 | YEVDTVLR | 497.76 | 2 | 20 |
| B879 | 879-889 | ALHPEEDPEGR | 625.29 | 2 | 24 |
| N89 | 89-97 | QDLPILVAK | 498.81 | 2 | 18 |
| N145 | 145-165 | GIEDEQDLVPLEVTGVVFHYR | 805.75 | 3 | 18 |
| N184 | 184-193 | LSSAIIAAPR | 499.80 | 2 | 15 |
| N194 | 194-214 | HLQAAFEDGFDNCDAGWLSDR | 808.68 | 3 | 15 |
| N257 | 257-269 | ELGGEVFYVGPAR | 697.36 | 2 | 18 |
| N316 | 316-322 | YPIQTPR | 437.74 | 2 | 22 |
| N582 | 582-589 | APVLELEK | 449.77 | 2 | 18 |
| N1155 | 1155-1170 | DFQWTDNTGLQFENWR | 1028.9 | 2 | 22 |
| N1195 | 1195-1209 | WNDVPCNYNLPYVCK | 971.44 | 2 | 28 |
| N1234 | 1234-1241 | YNVHATVR | 480.26 | 2 | 20 |
| N1242 | 1242-1257 | YQCNEGFAQHHVATIR | 644.30 | 3 | 18 |

^a^ – precursor *m/z* of the tryptic peptide

^b^ – normalized collision energy

Underlined C (C) indicates carbamidomethyled cysteine.

**Supplementary figure 1** Concentrations of brevican/neurocan peptides for lumbar and ventricular CSF (before and after shunt surgery).


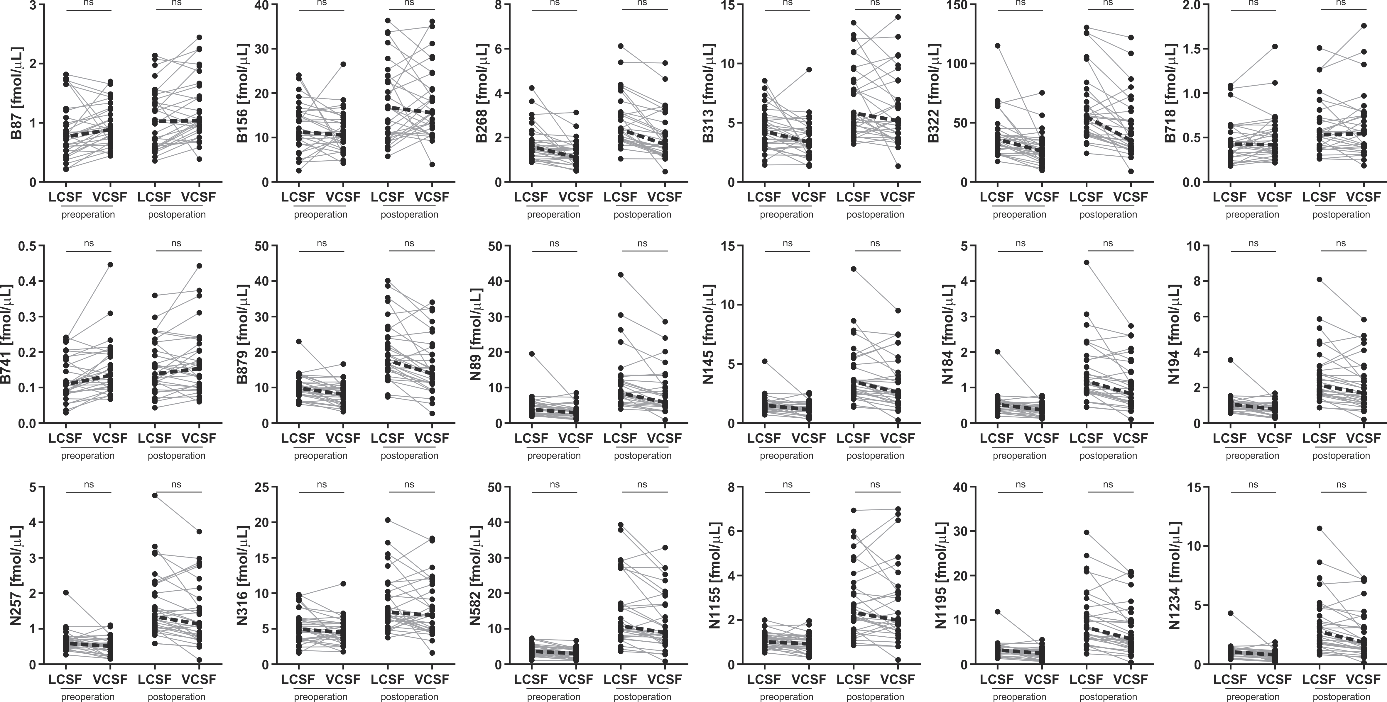


Brevican and neurocan peptide concentrations did not differ between ventricular or lumbar CSF (neither before nor after shunt surgery). Friedman test with Dunn’s multiple comparisons and Bonferroni correction for multiple testing were applied to investigate the differences between the lumbar and ventricular CSF. Dotted line represents change in median.

B: brevican; N: neurocan. The following number is the position of the first amino acid of the peptide. LCSF: lumbar CSF; VCSF: ventricular CSF.

**Supplementary figure 2.** Correlations between brevican and neurocan peptides in CSF before (A) and after (B) shunt surgery.


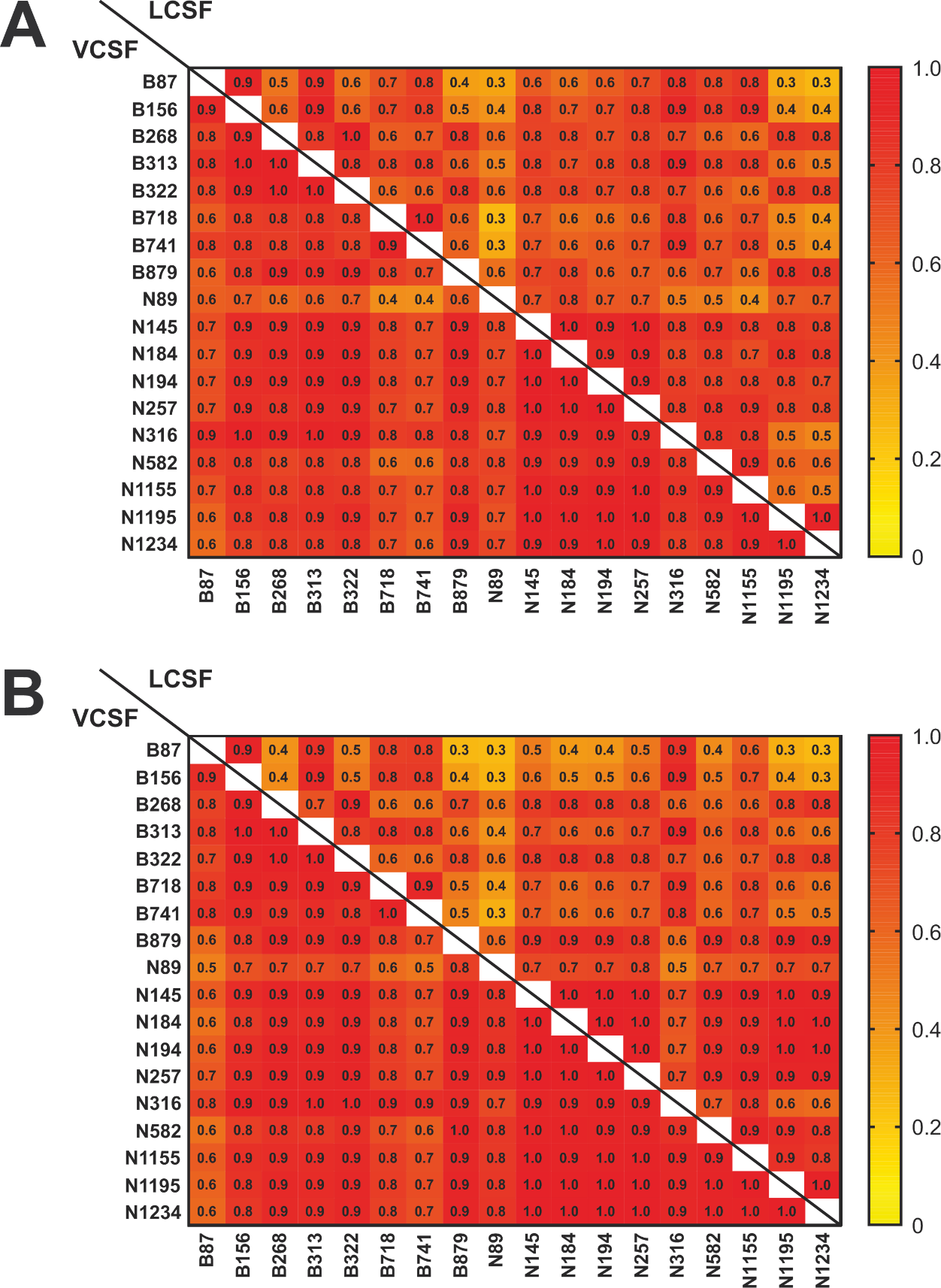


There was no clear change in brevican/neurocan fragmentation patterns before and after shunt surgery. B: brevican; N: neurocan. The following number is the first amino acid of the position of the peptide. LCSF: lumbar CSF; VCSF: ventricular CSF. The correlation matrix displays Spearman’s correlation coefficients (rho). Yellow indicates no correlation and the darker and more red the box, the closer the correlation is to positive 1.

**Supplementary figure 3** CSF concentrations of brevican and neurocan peptides pre- and postoperative (in lumbar and ventricular CSF).


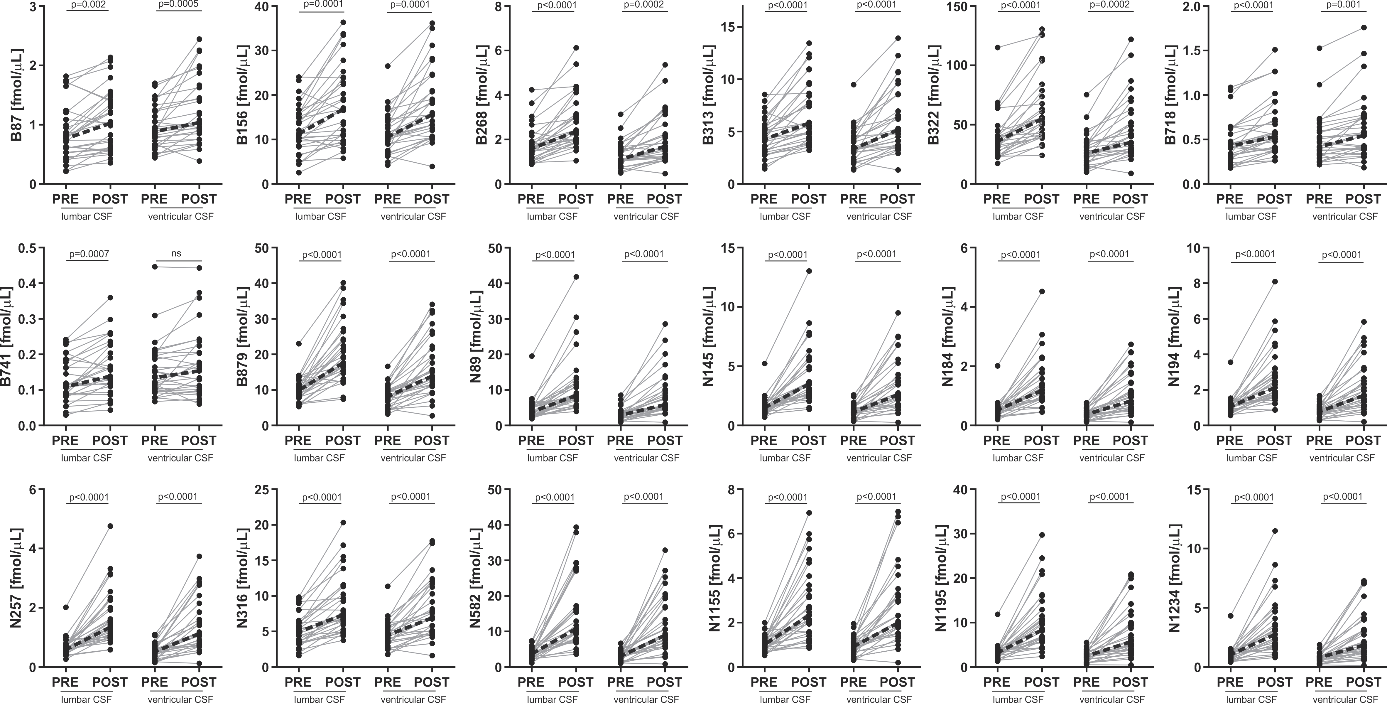


Brevican and neurocan peptide levels increased in both lumbar and ventricular CSF following shunt surgery compared with preoperative levels. Friedman test with Dunn’s multiple comparisons and Bonferroni correction for multiple testing were applied to investigate the differences before (``PRE``) and after (``POST``) shunt surgery. Dotted line represents change in median.

B: brevican; N: neurocan. The following number is the position of the first amino acid of the peptide.

**Supplementary figure 4** Ventricular, preoperative CSF concentrations of brevican and neurocan peptides in relation to cardiovascular disease comorbidity.

The majority of brevican and neurocan peptide levels were elevated in patients who suffered from cardiovascular comorbidity compared with patients without. Mann-Whitney test was applied to investigate the differences between the patients who did (CVD+; n=9) or did not (CVD-; n=22) suffer from cardiovascular disease (CVD). The two groups were age- and gender-matched. B: brevican; N: neurocan. The following number is the position of the first amino acid of the peptide.

**Supplementary figure 5** Ventricular, preoperative CSF concentrations of ECM proteins in relation to diabetes.

Diabetes did not show any significant effect on the ECM protein concentrations in CSF. Mann-Whitney test was applied to investigate the differences between the patients who did (diabetes+; n=13) and did not (diabetes-; n=18) suffer from diabetes. The two groups were age-and gender-matched. A representative peptide of brevican (B156) and of neurocan (N194) has been displayed.

**Supplementary figure 6** Ventricular, preoperative CSF concentrations of ECM proteins in relation to hypertension.


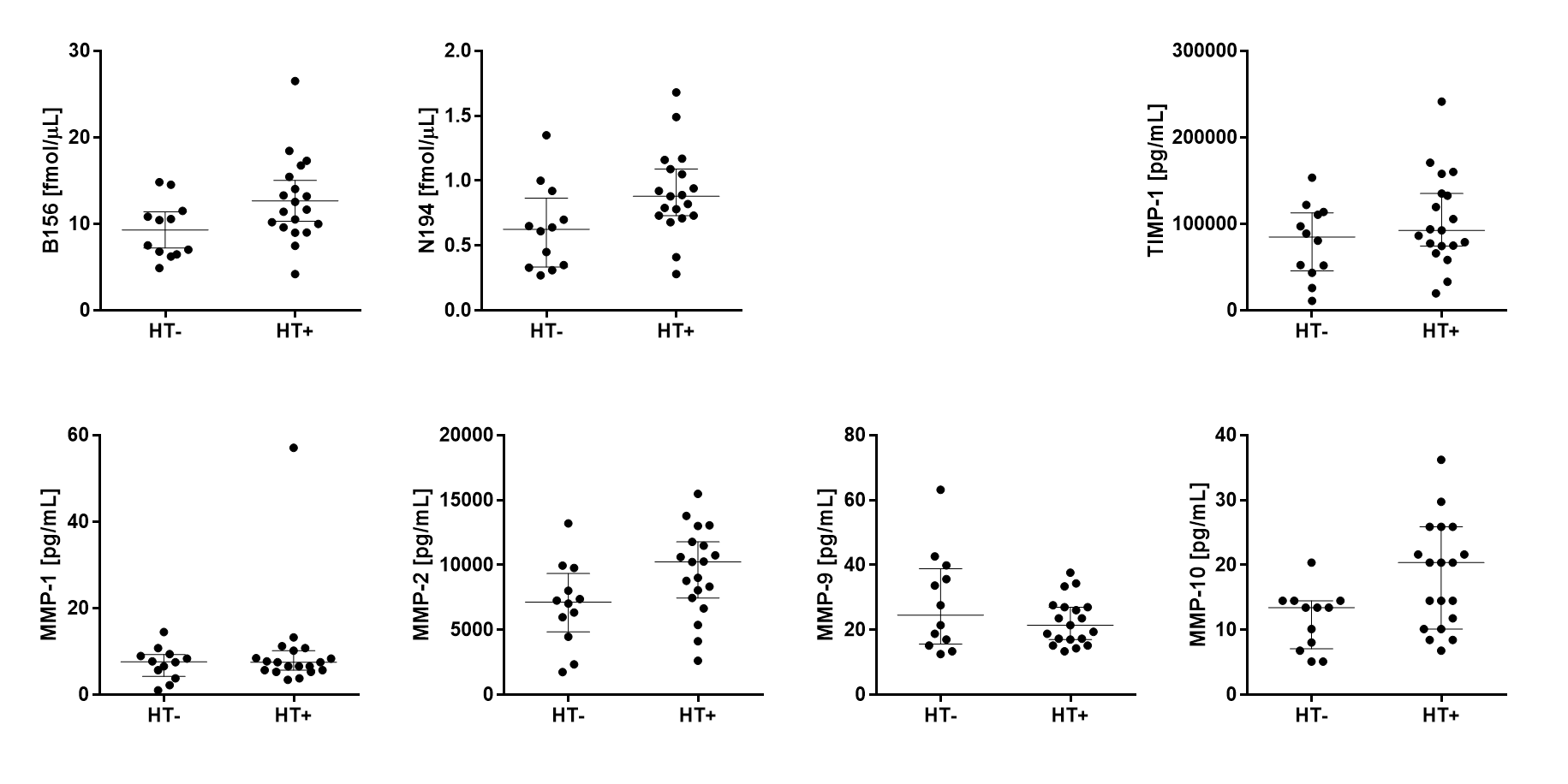


Hypertension did not have any significant effect on the ECM protein concentrations in CSF, Mann-Whitney test was applied to investigate the differences between the patients who did (HT+; n=19) and did not (HT-; n=12) suffer from hypertension. The two groups were age- and gender-matched. A representative peptide of brevican (B156) and of neurocan (N194) has been displayed.

**Supplementary figure 7** Ventricular, preoperative CSF concentrations of ECM proteins regarding the number of vascular risk factors.


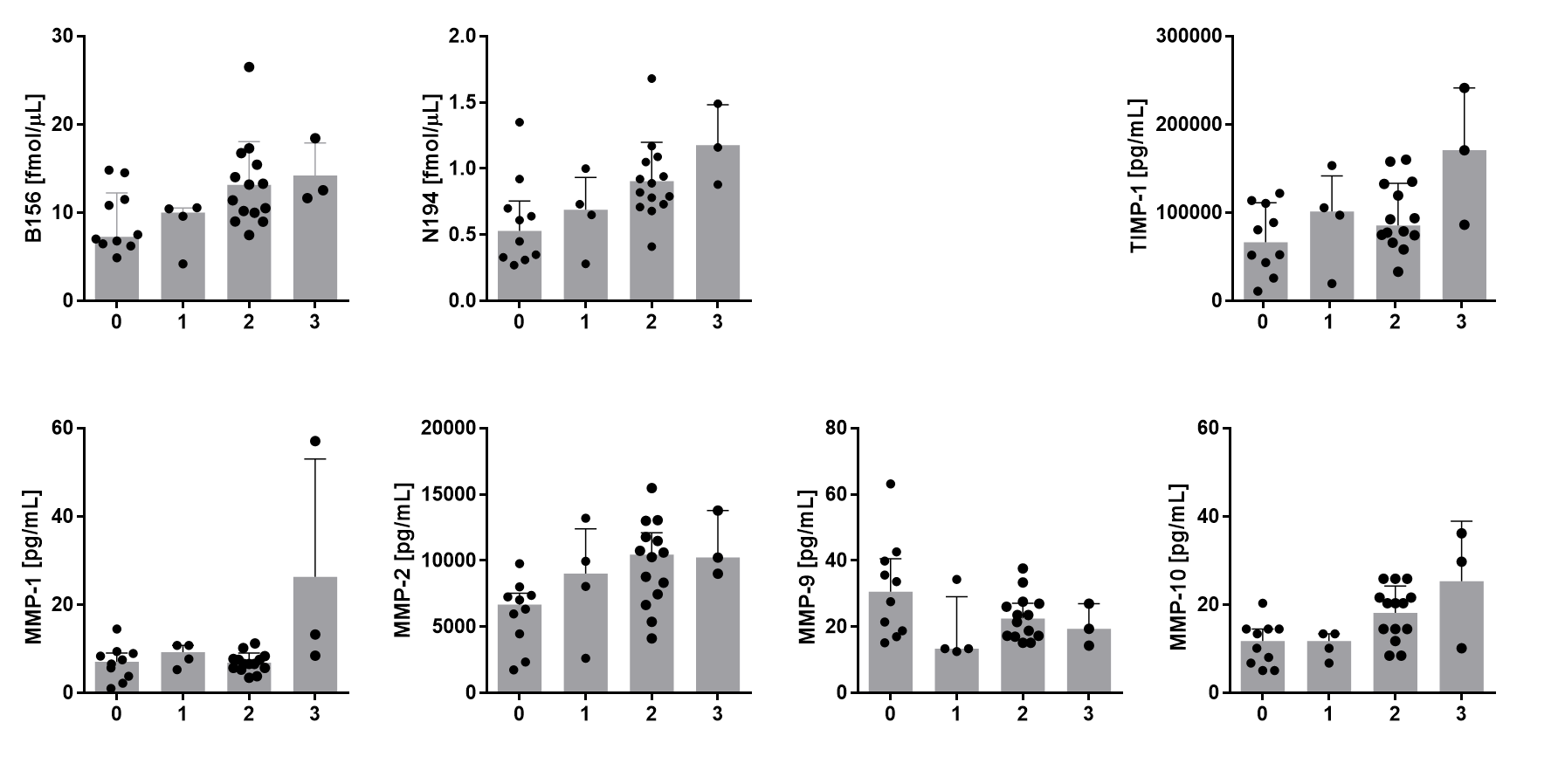


There was a gradual trend of brevican/neurocan levels to increase with the number of vascular risk factors present, although the change did not reach significance. Kruskal-Wallis test with Dunn’s multiple comparisons was applied to investigate the differences between the patients who had none, one, two or three of the following vascular risk factors: cardiovascular disease, diabetes and hypertension. There was a gradual trend of the majority of ECM protein levels to increase with the number of vascular risk factors present.
